# Efficacy and toxicity of three concurrent chemoradiotherapy regimens in treating nasopharyngeal carcinoma: Comparison among cisplatin, nedaplatin, and lobaplatin

**DOI:** 10.1097/MD.0000000000031187

**Published:** 2022-12-09

**Authors:** Xuexia Liang, Qiaodan Liu, Wei Yao, Shuai Yang

**Affiliations:** a Department of Cancer Center, the Fifth Affiliated Hospital of Sun Yat-sen University, Zhuhai, People’s Republic of China; b Guangdong Provincial Key Laboratory of Biomedical Imaging, Guangdong Provincial Engineering Research Center of Molecular Imaging, the Fifth Affiliated Hospital of Sun Yat-sen University, Zhuhai, People’s Republic of China; c Department of Head and Neck Oncology, the Fifth Affiliated Hospital of Sun Yat-sen University, Zhuhai, People’s Republic of China; d Department of Radiotherapy and Minimally Invasive Surgery, the Fifth Affiliated Hospital of Sun Yat-sen University, Zhuhai, People’s Republic of China

**Keywords:** cisplatin, efficacy, lobaplatin, nasopharyngeal carcinoma, nedaplatin, toxicity

## Abstract

Few studies directly compare efficacy and toxicity among lobaplatin, nedaplatin and cisplatin concurrently with intensity-modulated radiotherapy (IMRT) in nasopharyngeal carcinoma (NPC). Totally 141 treatment-naïve NPC without distant metastasis receiving IMRT concurrent with cisplatin or nedaplatin or lobaplatin were retrospectively enrolled. Their response rate, toxicity and long-term survival were compared. Complete response (CR) rates of concurrent lobaplatin (CR-nasopharynx [CR-nx], 82.7%; CR-cervical lymph node [CR-nd], 94.2%) were lower than those of cisplatin (CR-nx, 89.3%; CR-nd, 98.2%) and nedaplatin (CR-nx, 93.9%; CR-nd, 97.0%), but statistical significance wasn’t detected. Estimated five-year overall survival (OS), local relapse-free survival (LRFS), distant metastasis-free survival (DMFS) and progression-free survival (PFS) weren’t statistically significant between three groups. Multivariable analysis by COX proportional hazards model showed concurrent chemotherapy regimen wasn’t an independent prognostic factor. Gastrointestinal toxicity was prevalent in platinum-based concurrent chemotherapy; cisplatin group suffered heavier (≥grade 2) than other two groups. More nephrotoxicity happened in cisplatin group (17.9%) than nedaplatin (9.1%) and lobaplatin (2.0%) groups. Incidence of dermatitis of ≥grade 2 was higher in cisplatin group (60.7%) than nedaplatin (27.3%) and lobaplatin (9.6%) groups. More patients in lobaplatin and nedaplatin groups suffered from any grade thrombocytopenia (*P* < .001), but incidence of severe thrombocytopenia (≥grade 3) was similar. Economic cost was significant less in lobaplatin group. Cisplatin, nedaplatin and lobaplatin are equally effective when used concurrently with IMRT in NPC. Lobaplatin and nedaplatin have potential to be alternatives to cisplatin in terms of less severe acute side-effects and economic cost.

## 1. Introduction

Nasopharyngeal carcinoma (NPC) originates from stratified squamous epithelium of nasopharynx and is an uncommon and unique type of head and neck cancer. NPC is prevalent in southeast Asia (especially southern China), eastern Asia and north Africa. Epstein-Barr virus infection is the proven pathogenesis. More than 95% of NPC cases are diagnosed as non-keratinizing subtype in endemic area. NPC has strong propensity to metastasize to regional cervical lymph nodes and distant organs. NPC is highly sensitive to chemo-radiotherapy.^[1]^ Cisplatin-based chemo-radiotherapy is recommended as standard treatment according to National Comprehensive Cancer Network (NCCN) guideline. Several trials indicated other platinum drugs (carboplatin^[2–4]^ or nedaplatin^[5–7]^) seemed to be alternatives to cisplatin in concurrent chemo-radiotherapy setting because carboplatin or nedaplatin had less severe side-effects (nausea and vomiting, kidney and ear toxicity, and neurotoxicity) and did not need hydration for renal protection. But the non-inferiority when comparing these platinum drugs to cisplatin was still undetermined.^[4,8]^ Lobaplatin-based chemotherapy plus definitive intensity-modulated radiotherapy (IMRT) has been reported^[9–12]^ in single-arm studies. But efficacy and toxicity of nedaplatin, lobaplatin and cisplatin haven’t been directly compared in concurrent chemo-radiotherapy in a study due to lack of head-to-head comparative trial. This retrospective study was done to compare short-term response rate, toxicity and long-term survival of cisplatin, nedaplatin and lobaplatin in concurrent chemotherapy plus IMRT in NPC.

## 2. Methods and Materials

### 2.1. Patient enrollment and evaluation

IMRT had been implemented since July 1, 2014 in the Fifth Affiliated Hospital of Sun Yat-sen University. The enrollment criteria were as follow: newly-diagnosed and histopathologically proven NPC without distant metastasis; Eastern Cooperative Oncology Group score ≤ 1; had received definitive IMRT plus platinum-based concomitant chemotherapy; had refused neoadjuvant or adjuvant chemotherapy. pretreatment evaluation included history, physical examination, optic fiber nasopharyngoscopy, enhanced magnetic resonance imaging (MRI) or computerized tomography (CT) scanning of nasopharynx and neck, CT plain scanning of chest and upper abdomen, bone scanning (by emission computed tomography), electrocardiography, a complete blood count and a biochemical profile. Enhanced CT scanning of chest and abdomen or whole body ^18^F-fluorodeoxyglucose positron emission tomography/computerized tomography or even biopsy was done for workup of potential distant-metastasis. Plasma Epstein-Barr virus deoxyribonucleic acid measured by quantitative polymerase chain reaction test was optional. Restaging was done by revising pretreatment evaluation according to the 8^th^ edition clinical classification of American Joint Committee on Cancer staging system.

### 2.2. Treatment

Concurrent chemotherapy was given synchronized with definitive radiotherapy. According to treatment practice for NPC during the study period in our hospital, there were three concomitant chemotherapy schemes. Concurrent cisplatin was intravenously infused at a dose of 80 mg/m^2^ on day 1, 22, 43. Prophylactic hydration and diuretics were administered to reduce nephrotoxicity. Concurrent nedaplatin was intravenously administered at a dose of 80 mg/m^2^ on day1, 22, 43. Concomitant lobaplatin was intravenously infused at a dose of 50 mg/m^2^ on day 1, 22, 43. Chemotherapy given within one week after radiotherapy was also defined as a concomitant one. Antiemetic drugs, such as 5-HT3 receptor antagonists (tropisetron 4.5 mg every 12 hours or palonosetron 0.3 mg every 48 hours, intravenously), dexamethasone (10 mg intravenously) and metoclopramide (10mg intramuscularly), were prophylactically used to relieve chemotherapy-induced nausea and vomiting.

Definitive radiotherapy was performed utilizing high energy 6 MV X-ray produced by linear accelerators (Elekta Synergy or Infinity, Elekta). All patients received IMRT by simultaneously integrated boost technique, with 2.12 Gy per fraction and total dose of 70 Gy for gross tumor volume in nasopharynx. Every patient was irradiated once a day from Monday to Friday. Details on implementing IMRT were written in Appendix 1, Supplemental Digital Content, http://links.lww.com/MD/H662 on radiotherapy and Figure S1, Supplemental Digital Content, http://links.lww.com/MD/H663.

### 2.3. Follow-up and assessment of toxicities and efficacy

After concomitant chemoradiotherapy, patients were observed every three months during the first three years, and every six months during the fourth to fifth years, and once a year thereafter. Regular follow-up examinations included physical examination and nasopharyngoscopy. MRI or CT of the nasopharynx and neck was used to evaluate efficacy every six months. Local relapse and distant metastasis were confirmed by continuous dynamic observation of MRI/positron emission tomography/computerized tomography imaging or biopsy. Efficacy was assessed according to the Response Evaluation Criteria in Solid Tumor version 1.1. Toxicity was graded by National Cancer Institute Common Terminology Criteria for Adverse Events version 4.0. Toxicities of ≥ grade 3 were defined as high grade. Late toxicity was defined as those arising from the fourth month on after chemoradiotherapy.

### 2.4. Statistical analysis

SPSS software (version 16.0, SPSS, Chicago, IL, USA) was used to perform statistical analysis and two-tailed *P* values < 0.05 were considered statistically significant. Cross table with Chi-square test of independence was used to assess the homogeneity of distribution of patient demographics and clinical characteristics in different groups. Comparison of quantitative data was performed by one-way analysis of variance with *LSD-t* test. Cumulative survival rates were calculated by life table method, and univariate analysis for survivals was done by Kaplan–Meier method and log-rank test. Multivariate analysis was performed by the COX proportional hazards model to determine independent prognostic factors and to calculate hazard ratio (HR) and its 95% confidence index (CI). Terms of survival were defined as follow: local relapse-free survival (LRFS), time interval from completing chemoradiotherapy to the first-discovered local recurrence in irradiation field or the latest follow-up when censored; distant metastasis-free survival (DMFS), time interval from completing chemoradiotherapy to the first-discovered distant metastasis or the latest follow-up when censored; progression-free survival (PFS), time interval from finishing chemoradiotherapy to the first-discovered tumor recurrence at any site or the latest follow-up when censored; overall survival (OS), time interval from finishing chemoradiotherapy to death or the latest follow-up when censored.

## 3. Results

### 3.1. Patient characteristics and treatment compliance

From November 2014 to June 2018, there were 141 patients eligible for evaluation, who refused neoadjuvant or adjuvant chemotherapy and finally received only platinum-based concomitant chemoradiotherapy. Concurrent carboplatin chemotherapy was excluded because of very small sample size (only five patients received carboplatin-based concomitant chemoradiotherapy). The entire cohort had 91 (64.5%) men and 50 (35.5%) women. One hundred thirty-two patients (93.6%) were diagnosed as undifferentiated non-keratinized carcinoma. Median age of entire cohort was 48 years (range, 21–76 years). Eighty-eight patients (62.4%) had comorbidity. Median follow-up was 44.5 months (range, 3.7–78.9 months). Patients’ characteristics were summarized in Table 1, and Cross tables with Chi-square test of independence showed that distribution of patients’ demographics and clinical characteristics in different groups was homogeneous.

**Table 1:** Distribution of patient demographics and clinical characteristics.

Characteristics	Cisplatin group (n = 56)	Nedaplatin group (n = 33)	Lobaplatin group (n = 52)	*P* value
**Median age, years** (Range)	47.5 (26.0–74.0)	50.0 (32.0–76.0)	47.5 (21.0–76.0)	
***Gender***				0.329
Male	32	23	36	
Female	24	10	16	
***T stage*** *				0.099
T_1_	7	9	11	
T_2_	15	9	6	
T_3_	23	8	17	
T_4_	11	7	18	
***N stage*** *				0.057
N_0-1_	11	14	18	
N_2-3_	45	19	34	
***Clinical stage*** *				0.358※
Ⅱ	5	8	6	
Ⅲ	28	12	25	
Ⅳa	23	13	21	
***Histology***				0.736※
Keratinizing carcinoma	0	1	1	
Differentiated non-keratinizing carcinoma	2	2	3	
Undifferentiated non-keratinizing carcinoma	54	30	48	
***Comorbidity***				0.851※
Cardio-encephalo-vascular	10	7	10	
Respiratory	5	5	8	
Hepatobiliary and gastrointestinal	14	5	13	
Renal	2	3	3	
malignancy	5	1	1	
Autoimmune and metabolic	18	10	16	

* According to the 8^th^ edition clinical classification of American Joint Committee Cancer staging system.

※ Fisher’s exact probability method.

(1) Cardio-encephalo-vascular diseases include acute cerebral infarction, arrhythmia, hypertension, coronary heart disease, deep venous thrombosis and viral meningitis.

(2) Renal diseases include kidney calculus, nephrotuberculosis and renal insufficiency.

(3) Hepatobiliary and gastrointestinal diseases include hepatitis B/C virus-related cirrhosis, liver enzyme elevation, steatohepatitis, peptic ulcer and viral hepatitis.

(4) Respiratory diseases include chronic obstructive pulmonary disease, pulmonary tuberculosis, pneumonia and chronic lung abscess.

(5) Autoimmune and metabolic diseases include diabetes, hyperlipemia, hyperthyroidism, dermatomyositis and hyperuricemia.

Forty-five (80.4%), twenty-five (75.8%) and forty (76.9%) patients completed three cycles of concurrent cisplatin, nedaplatin and lobaplatin, respectively; and eight (14.3%), seven (21.2%), and ten (19.2%) patients had two cycles; and three (5.4%), one (3.0%) and two (3.8%) patients only completed one cycle. The mean cumulative dose of cisplatin, nedaplatin and lobaplatin was 220 (91.7% of pre-planned dose), 218 (90.8% of pre-planned dose) and 136 (91.0% of pre-planned dose) mg/m^2^, respectively. No significance in chemotherapy compliance was detected (*P* = .912).

### 3.2. Efficacy

**Figure 1.**
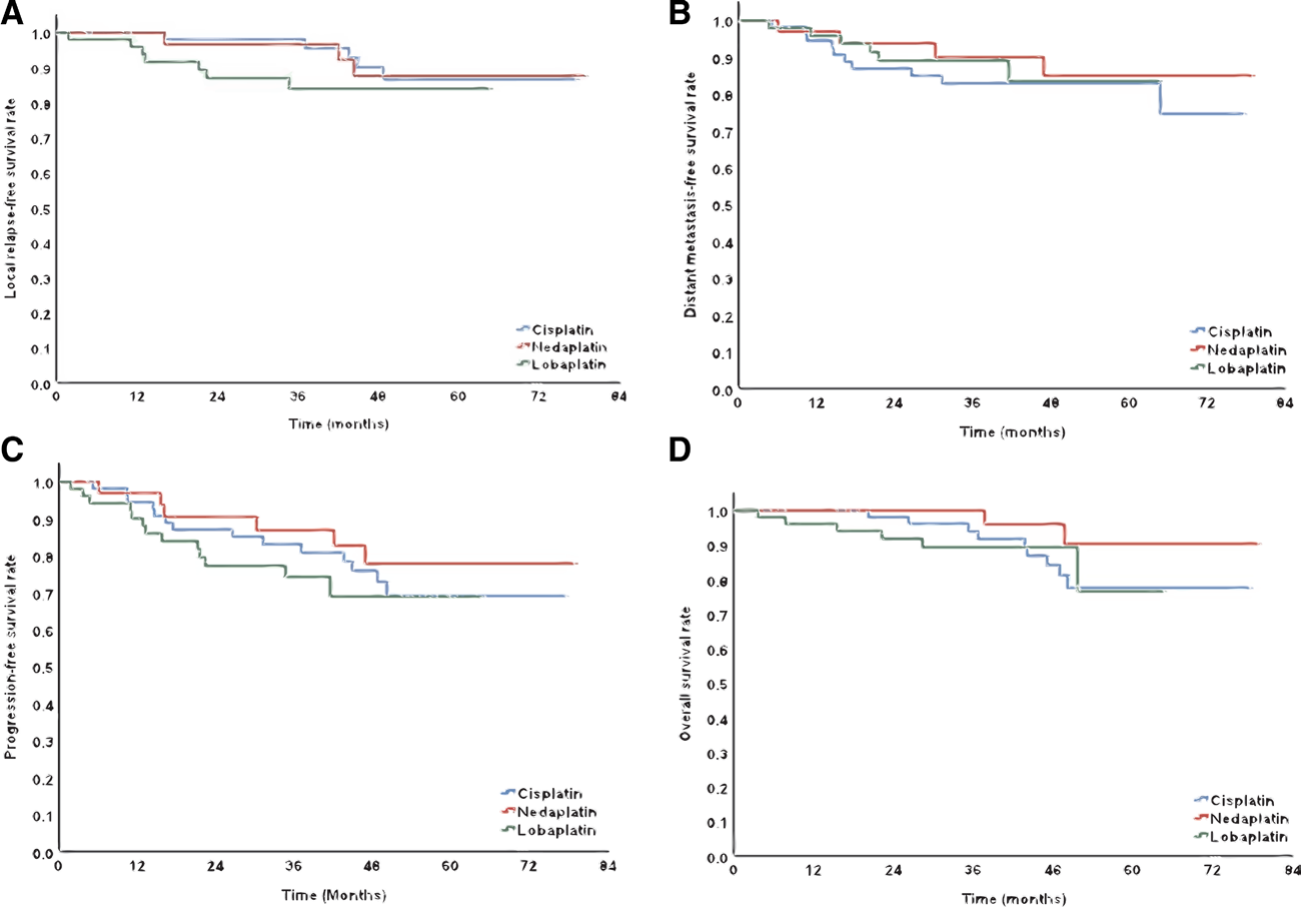
No significance in local relapse-free survivals (1A), distant metastasis-free survivals (1B), progression-free survivals (1C) or overall survivals (1D) between three platinum-based concurrent chemoradiotherapy regimens was observed.

Efficacy of 141 patients was listed in Table 2. Complete response rates of primary tumor in nasopharynx (CR-nx) were 89.3% (50/56), 93.9% (31/33), and 82.7% (43/52) in cisplatin, nedaplatin and lobaplatin groups (*P* = .277), respectively. Complete response rates of cervical lymph node metastasis (CR-nd) were 98.2% (55/56), 97.0% (32/33), and 94.2% (49/52) in cisplatin, nedaplatin and lobaplatin groups (*P* = .526), respectively. The median follow-up time of cisplatin, nedaplatin and lobaplatin groups were 49.1 (interquartile range [IQR] 36.5–60.0), 49.7 (IQR 37.2–56.3) and 36.6 (IQR 25.4.0–47.5) months, respectively. Local recurrence happened to ten patients (four in cisplatin group, two in nedaplatin group and four in lobaplatin group, respectively). One patient in cisplatin group and three patients in lobaplatin group had nodal relapse. There were twenty distant metastasis cases (ten in cisplatin group, four in nedaplatin group and six in lobaplatin group). Details about metastasis sites was provided in Table S1, Supplemental Digital Content, http://links.lww.com/MD/H664. Seventeen patients died (nine in cisplatin group, two in nedaplatin group and six in lobaplatin group), and one patient in lobaplatin group died from acute severe nasopharyngeal hemorrhage four months after chemoradiotherapy. The estimated five-year LRFS, DMFS, PFS OS rates were 86% (cisplatin) vs 88% (nedaplatin) vs 84% (lobaplatin), 74% (cisplatin) vs 86% (nedaplatin) vs 84% (lobaplatin), 68%(cisplatin) vs 79% (nedaplatin) vs 70% (lobaplatin), 77%(cisplatin) vs 88% (nedaplatin) vs 77% (lobaplatin), respectively. Log-rank test showed no significant difference between LRFS, DMFS, PFS and OS curves in three regimens (Fig. 1). In multivariate analysis, T4 stage was the only independent adverse prognostic factor of LRFS for whole cohort (HR, 1.086; 95% CI, 1.014–1.163; *P* =  .019). Early N stage (N_0-1_) was the only independent favorable prognostic factor of DMFS for entire cohort (HR, 2.404; 95% CI, 1.109–5.212; *P* =  .026). Concomitant chemotherapy regimen didn’t significantly influence short-term and long-term efficacy of NPC here.

**Table 2:** Short-term response and long-term efficacy of 141 NPC patients receiving IMRT concurrent with three platinum chemotherapy regimens.

Treatment group	Short-term efficacy*				Long-term efficacy					
	Primary tumor		Lymph node metastasis		Five-year survival rates (%)				Survival interval# (months)	
	CR	PR	CR	PR	LRFS	DMFS	PFS	OS	PFS	OS
Cisplatin	50	6	55	1	86	74	68	77	62.8 ± 3.4 (56.1–69.5)	69.4 ± 2.5 (64.5–74.2)
Nedaplatin	31	2	32	1	88	86	79	88	67.7 ± 4.1 (59.7–75.8)	75.6 ± 2.2 (71.2–79.9)
Lobaplatin	43	9	49	3	84	84	70	77	50.8 ± 3.3 (44.3–57.3)	57.8 ± 2.6 (52.7–62.9)

CR = complete response, DMFS = distant metastasis-free survival, IMRT = intensity-modulated radiotherapy, LRFS = local relapse-free survival, NPC = nasopharyngeal carcinoma, OS = overall survival, PFS = progression-free survival, PR = partial response.

### 3.3. Toxicities

**Figure 2.**
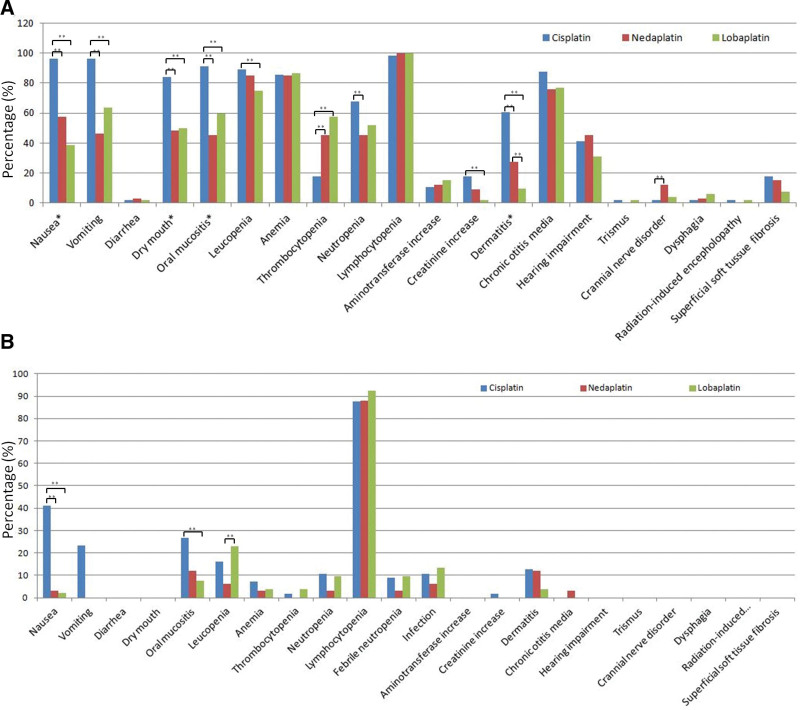
Lobaplatin and nedaplatin caused less and milder toxicities. Toxicities of ≥ grade 1 (Fig. 2A) and ≥ grade 3 (Fig. 2B) in three platinum-based concomitant chemoradiotherapy regimens. *, ≥grade 2; **, *P* < .05.

All patients were eligible for toxicity assessment. The whole cohort had nausea, oral mucositis and dry mouth. Patients in cisplatin group suffered heavier than those in nedaplatin and lobaplatin groups when frequencies of toxicities of ≥ grade 2 and ≥ grade 3 were compared (Table 3 and Figure 2). Higher frequency of any grade vomiting was recorded in cisplatin group (96%) than other two groups (nedaplatin group, 79%; lobaplatin group, 64%). None of nedaplatin group or lobaplatin group suffered from vomiting of ≥ grade 3. Incidence of diarrhea was quite low. Any grade leucopenia in lobaplatin group (75%) was significantly less than that of cisplatin (89%) group. Leucopenia of ≥ grade 3 in lobaplatin group (23%) was more than that of nedaplatin (6%) group. Incidence of any grade thrombocytopenia was higher in nedaplatin group (45%) and lobaplatin group (58%) than that in cisplatin group (19%). Thrombocytopenia of ≥ grade 3 was similar among three groups. Nedaplatin led to less neutropenia of any grade (45%) comparing to cisplatin (68%). Occurrence and severity of anemia, lymphocytopenia, febrile neutropenia and infection was similar among three groups. Hepatotoxicity (manifested as aminotransferase increase) was mild and comparable between three groups. More nephrotoxicity (manifested as creatinine increase) of any grade happened in cisplatin (18%) group than nedaplatin (9%) and lobaplatin (2%) groups. No nephrotoxicity of ≥ grade 3 was reported. All patients suffered from acute dermatitis. Incidence of dermatitis of ≥ grade 2 decreased gradually in cisplatin (61%), nedaplatin (27%) and lobaplatin (10%) groups. There was more cranial nerve disorder in nedaplatin (12%) group compared to cisplatin (2%) group (*P* = .041). No significant difference of incidence of other late toxicities (such as chronic otitis media, hearing impairment, trismus, dysphagia, radiation-induced encephalopathy and superficial soft tissue fibrosis) was noticed. To sum up, cisplatin concomitant chemotherapy brought about heavier acute alimentary canal toxicity and dermatitis, and more leucopenia, neutropenia and nephrotoxicity, but fewer thrombocytopenia.

**Table 3:** Acute and chronic side-effects.

**Acute toxicities** #	Cisplatin (n = 56)				Nedaplatin (n = 33)				Lobaplatin (n = 52)				*P value* *(≥grade 1*)	*P value* *(≥grade 3*)
	Grade 1	Grade2	Grade3	Grade4	Grade1	Grade2	Grade3	Grade4	Grade1	Grade2	Grade3	Grade4		
***Digestive tract***														
Nausea	2 (4%)	31 (55%)	21 (38%)	2 (4%)	14 (42%)	18 (55%)	1 (3%)	0	32 (62%)	19 (37%)	1 (2%)	0	<0.001*	<0.001
Vomiting	13 (23%)	28 (50%)	13 (23%)	0	24 (73%)	2 (6%)	0	0	29 (56%)	4 (8%)	0	0	<0.001	<0.001※
Diarrhea	1 (2%)	0	0	0	1 (3%)	0	0	0	1 (2%)	0	0	0	1.000※	-
Dry mouth	9 (16%)	47 (84%)	0	0	17 (52%)	16 (48%)	0	0	26 (50%)	26 (50%)	0	0	<0.001*	-
Oral mucositis	5 (9%)	36 (64%)	15 (27%)	0	18 (55%)	11 (33%)	3 (9%)	1 (3%)	21 (40%)	27 (52%)	4 (8%)	0	<0.001*	0.021
***Hematological***														
Leucopenia	9 (16%)	32 (57%)	9 (16%)	0	8 (24%)	18 (55%)	2 (6%)	0	8 (15%)	19 (37%)	12 (23%)	0	0.135	0.117
Anemia	37 (66%)	7 (13%)	4 (7%)	0	27 (82%)	0	1 (3%)	0	35 (67%)	8 (15%)	2 (4%)	0	1.000※	0.695※
Thrombocytopenia	7 (13%)	2 (4%)	1 (2%)	0	12 (36%)	3 (9%)	0	0	19 (37%)	9 (17%)	2 (4%)	0	<0.001	0.615※
Neutropenia	16 (29%)	16 (29%)	5 (9%)	1 (2%)	6 (18%)	8 (24%)	1 (3%)	0 (%)	8 (15%)	14 (27%)	3 (6%)	2 (4%)	0.081	0.471※
Lymphocytopenia	0	7 (13%)	37 (66%)	12 (21%)	0	4 (12%)	27 (82%)	2 (6%)	0	4 (8%)	39 (75%)	9 (17%)	-	0.733※
***Febrile neutropenia***	0	0	5 (9%)	0	0	0	1 (3%)	0	0	0	5 (10%)	0	-	0.571※
***Infection***	0	0	6 (11%)	0	0	0	2 (6%)	0	0	0	7 (13%)	0	-	0.612※
***Aminotransferase increase***	5 (9%)	1 (2%)	0	0	3 (9%)	1 (3%)	0	0	8 (15%)	0	0	0	0.769※	-
***Creatinine increase***	9 (16%)	0	1 (2%)	0	3 (9%)	0	0	0	1 (2%)	0	0	0	0.019※	-
***Dermatitis***	22 (39%)	27 (48%)	7 (13%)	0	24 (73%)	5 (15%)	3 (9%)	1 (3%)	47 (90%)	3 (6%)	2 (4%)	0	<0.001*	0.218※
**Late toxicities** # ξ														
Chronic otitis media	40 (71%)	9 (16%)	0	0	20 (61%)	4 (12%)	1 (3%)	0	32 (62%)	8 (15%)	0	0	0.263	-
Hearing impairment	22 (39%)	1 (2%)	0	0	14 (42%)	1 (3%)	0	0	11 (21%)	5 (10%)	0	0	0.342	-
Trismus	1 (2%)	0	0	0	0	0	0	0	1 (2%)	0	0	0	1.000※	-
Crannial nerve disorder	1 (2%)	0	0	0	4 (12%)	0	0	0	2 (4%)	0	0	0	0.097※	-
Dysphagia	1 (2%)	0	0	0	1 (3%)	0	0	0	3 (6%)	0	0	0	0.636※	
Radiation-induced encephalopathy	1 (2%)	0	0	0	0	0	0	0	1 (2%)	0	0	0	1.000※	-
Superficial soft tissue fibrosis	10 (18%)	0	0	0	5 (15%)	0	0	0	4 (8%)	0	0	0	0.277※	-

# According to the National Cancer Institute Common Terminology Criteria for Adverse Events version 4.0.

*≥ grade 2.

ξ Adverse events which arised 90 days after chemoradiotherapy are defined as late toxicities.

※ Fisher’s exact probability method.

### 3.4. Economic assessment

The lobaplatin group required shorter hospitalization (58.71 ± 7.85 days) compared to other two (nedaplatin group: 61.79 ± 14.63 days; cisplatin group: 62.21 ± 6.38 days), although no statistical significance was displayed (Fig. S2A, Supplemental Digital Content, http://links.lww.com/MD/H665). Hospitalization cost of lobaplatin group (RMB￥ 9.60 ± 1.88 × 10^4^) was significantly less than those of cisplatin group (RMB￥ 10.86 ± 1.32 × 10^4^) and nedaplatin group (RMB￥ 10.53 ± 1.49 × 10^4^) (Fig. S2A, Supplemental Digital Content, http://links.lww.com/MD/H665).

## 4. Discussion

To our knowledge, efficacy and toxicity of lobaplatin, nedaplatin and cisplatin haven’t been directly compared in concomitant radio-chemotherapy. This research showed three platinum-based concomitant chemotherapy regimens plus IMRT achieved similar short-term response and long-term survival. Compared to cisplatin, lobaplatin and nedaplatin caused milder digestive tract adverse reactions, fewer renal function abnormality, and milder dermatitis. Nedaplatin and lobaplatin caused more thrombocytopenia of any grade. Incidence of severe thrombocytopenia (≥grade 3) was similar. Lower toxicity might be the reason of less hospitalization economic burden.

Previous studies had indicated the anti-cancer activity of lobaplatin in NPC.^[9–14]^ Lobaplatin achieved objective response rate (ORR) of 83% to 100% in neoadjuvant chemotherapy in treatment-naïve NPC.^[9,11,12]^ The ORR of lobaplatin was 64% to 67% in palliative chemotherapy in recurrent or metastatic NPC who had failed other platinum-based chemotherapy.^[13,14]^ This study reported an ORR of 100% after concomitant lobaplatin-IMRT without neoadjuvant chemotherapy, coincident with data from previous studies^[9,11,12]^ with neoadjuvant chemotherapy. Five-year estimated survival rates of concurrent lobaplatin were comparable to those of concomitant cisplatin and nedaplatin. In literature, short-term (CR rate) and long-term (3–5-year PFS) efficacy of lobaplatin-based chemo-radiotherapy was similar to those of cisplatin-based regimen.^[15–17]^ Concomitant nedaplatin-IMRT also produced parallel short-term response and long-term effect to those of cisplatin-IMRT in this study. According to Tang LQ et al and Tang C et al, nedaplatin exhibited similar efficacy compared to cisplatin in concomitant chemotherapy setting with or without neoadjuvant chemotherapy.^[6,7]^ There is no study directly comparing effectiveness between lobaplatin and nedaplatin except ours. Response rates and estimated survival rates of lobaplatin-IMRT and nedaplatin-IMRT did not significantly differ in our study. To sum up, efficacy of lobaplatin, nedaplatin and cisplatin was similar when used concurrently with IMRT in treating NPC.

The other important goal of NPC treatment was to reduce toxicities. Improvement in image technology and irradiation technique has facilitated accurate target delineating, dose delivery and normal organ sparing and mitigated radiotherapy-related toxicities.^[18]^ Platinum was the most widely used drug for NPC in induction, concomitant and adjuvant chemotherapy settings. The first-generation platinum, cisplatin, was established as standard agent according to NCCN guideline since randomized controlled trials adopting cisplatin-based regimens reported evident survival benefit.^[19,20]^ Cisplatin was associated with more side-effects, including nausea, vomiting and renal toxicity. To protect kidney, hydration was essential for high dose cisplatin-based chemotherapy. Cisplatin might be relatively contra-indicant for patients with renal or cardiac dysfunction. Therefore, nedaplatin or lobaplatin or reduced dosage of cisplatin were chosen for patients with comorbidity (accounting for 62.4% of study cohort in this study) or advanced age here.

Incidences of vomiting and nausea of ≥ grade 2 and ≥ grade 3 were higher in cisplatin group even cisplatin dose was reduced by 20% (NCCN guideline recommends 100 mg/m^2^ in systemic therapy/radiotherapy). Cisplatin seemed to aggravate radiotherapy-related acute toxicity. Cisplatin caused more oral mucositis of ≥ grade 2 and ≥ grade 3, dry mouth of ≥ grade 2, and dermatitis of ≥ grade 2 than nedaplatin and lobaplatin, consistent with previous studies.^[6,7]^ Although nedaplatin and lobaplatin cause more thrombocytopenia (any grade), consistent with previous study,^[7,11,21]^ incidence of ≥ grade3 thrombocytopenia between groups was similar in this study, which tallied with a study from Tang LQ et al^[7]^ Cisplatin led to more neutropenia than nedaplatin (*P* = .038) and lobaplatin, which was similar to result from Lv X et al^[16]^ but contrary to that from Tang C et al^[6]^ More leucopenia was observed in cisplatin group, which was consistent with Lv X et al’s result.^[16]^ The renal function impairment (manifested as creatinine increase) in nedaplatin and lobaplatin groups was less than that in cisplatin group, similar to reports from Cheng Y et al and Shukuya T et al^[21,22]^ All mentioned above indicated that lobaplatin and nedaplatin decreased acute side-effects.

NPC patients in this study refused induction or adjuvant chemotherapy, partly due to comorbidity (62.4% of entire cohort) and old age (≥ 65 years, 9.9% of study cohort), which impaired tolerance of cisplatin or increased the risk of heart failure during hydration. Lobaplatin and nedaplatin were 70.72 and 13.57 folds more expensive than cisplatin, but overall cost of concomitant lobaplatin in hospitalization was significantly less than those of cisplatin and nedaplatin. Perhaps lower toxicity cut down the cost for supportive care (nausea, vomiting, mucositis, dermatitis, etc). The hospitalization length between three groups didn’t vary notably partly due to local health insurance policy, which covered more hospitalization fee and induced patients to complete concomitant chemo-radiotherapy course in hospitalization.

There are two limitations in this study. First, it is a retrospective clinical analysis whose evidence level is inferior to that of randomized controlled trial. And second, sample size here is not large enough. However, this is the first retrospective study directly comparing efficacy and toxicity of nedaplatin, lobaplatin and cisplatin. Our results showed that lobaplatin and nedaplatin were equally effective, and might be alternatives to cisplatin. And randomized controlled trial head-to-head comparing lobaplatin, nedaplatin and cisplatin is required to optimize concurrent chemotherapy regimen.

## 5. Conclusion

This study reported similar efficacy of three concurrent chemotherapy regimens. Lobaplatin and nedaplatin, could reduce severe acute side-effects of cisplatin without increasing economic burden.

## Author Contributions

Study conception and design (Xuexia Liang). Data acquisition (Xuexia Liang, Qiaodan Liu, Wei Yao and Shuai Yang). Data statistical analysis and interpretation (Xuexia Liang and Qiaodan Liu). Manuscript writing (Xuexia Liang and Qiaodan Liu). All authors contributed to this article and approved the submitted version.

**Conceptualization:** Xuexia Liang.

**Writing - original draft:** Xuexia Liang, Qiaodan Liu.

## Supplementary Material


