# Supplementary material for: Efficacy and toxicity of three concurrent chemoradiotherapy regimens in treating nasopharyngeal carcinoma: Comparison among cisplatin, nedaplatin, and lobaplatin
Source: Medicine (Baltimore). 2022 Dec 9;101(49):e31187. doi: 10.1097/MD.0000000000031187 (PMC9750602; doi:10.1097/MD.0000000000031187)
Supplement: Supplementary file 1 [file medi-101-e31187-s001.pdf]

The principle of intensity-modulated radiotherapy implementation

Intensity-modulated radiotherapy with simultaneously integrated boost (IMRT-SIB) technique was routinely applied in our cancer centre. Each patient was immobilized in supine position with a thermoplastic mask. Plain and enhanced CT scanning from 2cm above the superciliary arch to 2cm below the clavicle, with a 3mm slide thickness, were performed to every patient except existence of allergy to iodine contrast agent. IMRT-SIB plan was accomplished by nine coplanar 6MV X-Ray beams. Target delineation and dose distribution optimization were performed by Ocentra (from July 2014 to December 2016) or Raystation (from Oct 2014 to Feb 2017) or Monaco (from Jan 2017 to now) treatment planning system.

The gross tumor volume (GTV) includes GTVnx (the sum of primary tumor volume of nasopharynx and enlarged retropharyngeal lymph nodes) and GTVnd (clinically involved neck lymph nodes). Lymph nodes with ≥one of the following characteristics, which were indicated by CT or MR imaging, would be diagnosed as lymphatic metastasis.

1) diameter ≥1.5 cm for lymph nodes in level Ⅱ, Ⅲ and Ⅳ areas, or diameter ≥ 1.0 cm in other areas, or ratio of longer diameter to shorter diameter <2.

2) fused lymph nodes.

3) heterogeneous ring-enhancement.

4) irregular necrosis.

5)≥ 3 lymph nodes distributed in clusters in one area.

GTV delineation was based on MRI-CT fusion images and/or PET-CT (if available), nasopharyngoscopy and physical examination.

High-risk clinical target volume (CTV1, aim to encompass the high-risk sites of the microscopic invasion of the primary tumor) was defined as the sum volume of GTVnx plus a 5~10 mm margin (3~5 mm posteriorly if primary tumor was adjacent to brainstem or spinal cord). There were several extra details which should be noted when manually delineating CTV1.

1) the whole nasopharyngeal mucosa should be included in CTV1.

2) the top and bottom of CTV1 were at least two layers outside GTVnx.

3) CTV1 should include navicular fossa and oval foramen if parapharyngeal space was involved.

4) CTV1 should include carotid sheath if pharyngobasilar fascia was involved when no direct tumor involvement or lymphatic metastasis in carotid sheath.

5) CTV1 should cover posterior margins of ipsilateral internal jugular vein and contralateral internal carotid artery when only ipsilateral retropharyngeal lymph node metastasis was detected.

6) CTV1 should emcompass parapharyngeal space, medial pterygoid, navicular fossa and carotid sheath when pharyngobasilar fascia and retropharyngeal lymph node were concomitantly involved.

7) CTV1 should include soft tissue of bilateral foramen lacerum.

8) CTV1 should emcompass the base of sphenoid bone but not the bone cortex or medullary cavity of basilar clivus for T1N0 patients.

9) only bone cortex of basilar clivus was encompassed in CTV1 when primary tumor invaded musculus longus capitis without basilar clivus erosion.

10) If positive lymph node with extracapsular extention involving peripheral tissue exist, a high-risk clinical target volume, CTVnd, was delineated as the sum volume of GTVnd plus a 5 mm margin (Int J Radiat Oncol Biol Phys, 2006, 64(3):678-83.)

Low-risk clinical target volume (CTV2, aim to encompass the low-risk sites of the microscopic invasion of the primary tumor and positive lymph nodes) was defined as the sum volume of CTV1 plus a 5~10 mm margin (3~5 mm posteriorly if primary tumor was adjacent to brainstem or spinal cord) and cervical lymphatic drainage areas [including positive lymph node involved areas and the areas needing prophylactic irradiation]. There were several extra details which should be noted when manually delineating CTV2.

1)The bone cortex of basilar clivus was delineated as CTV2 when only nasopharyngeal mucosa was involved (T1 disease).

2)CTV2 should encompassed cavum medullare of clivus when primary tumor invaded musculus longus capitis without basilar clivus erosion.

3)The inferior 1/3~1/2 of sphenoid sinus, posterior ethmoid sinus, foramina of skull base, pterygopalatine fossa, posterior part of nasal cavity and posterior wall of maxillary sinus should be routinely included in CTV2.

4)CTV2 should gradually backwardly extend to cover the bilateral substernocleidomastoideus space (namely the level Ⅱlymphatic drainage area) when mastoid process and musculus digastricus appear.

5) Retropharyngeal lymph node area is excluded in CTV2 when hyoid bone appears.

6)If unilateral cervical lymph node metastasis is diagnosed, CTV2 should include lymphatic drainage areas of ipsilateral level Ⅱ, Ⅲ, Ⅳ, Ⅴa and Ⅴb and contralateral level Ⅱ, Ⅲand Ⅴa. If bilateral cervical lymph node metastasis exists, bilateral level Ⅱ, Ⅲ, Ⅳ, Ⅴa and Ⅴb should be delineated into CTV2.

7) Level Ⅰand level Ⅵshould not be routinely delineated in CTV2 except that presence of bulky enlargement and/or extracapsular invasion with submandibular gland involvement in level Ⅱa lymph node metastasis, and/or tumor invading peripheral structures whose lymphatic fluid is drained through level Ⅰa (e.g., oral structures) or level Ⅵ(e.g., trachea, larynx, thyroid gland) were confirmed.

Supplementary figure 1 demonstrates the relationships of GTVs and CTVs.
