# Supplementary material for: Efficacy and toxicity of three concurrent chemoradiotherapy regimens in treating nasopharyngeal carcinoma: Comparison among cisplatin, nedaplatin, and lobaplatin
Source: Medicine (Baltimore). 2022 Dec 9;101(49):e31187. doi: 10.1097/MD.0000000000031187 (PMC9750602; doi:10.1097/MD.0000000000031187)

2-Supplementary Figure 1 Inclusion relationships of target delineation. Dose prescription: GTVnx (gross tumor volume in nasopharynx): 70Gy; GTVnd (gross tumor volume of cervical lymph node metastasis): 64~70 Gy; CTV1 (clinical tumor volume of high-risk region) and CTVnd (clinical tumor volume of lymphatic drainage area with lymph node metastasis): 60Gy; CTV2 (clinical tumor volume of low-risk region): 54~56 Gy; Totally 33 fractions, one fraction per day, from Monday to Friday.

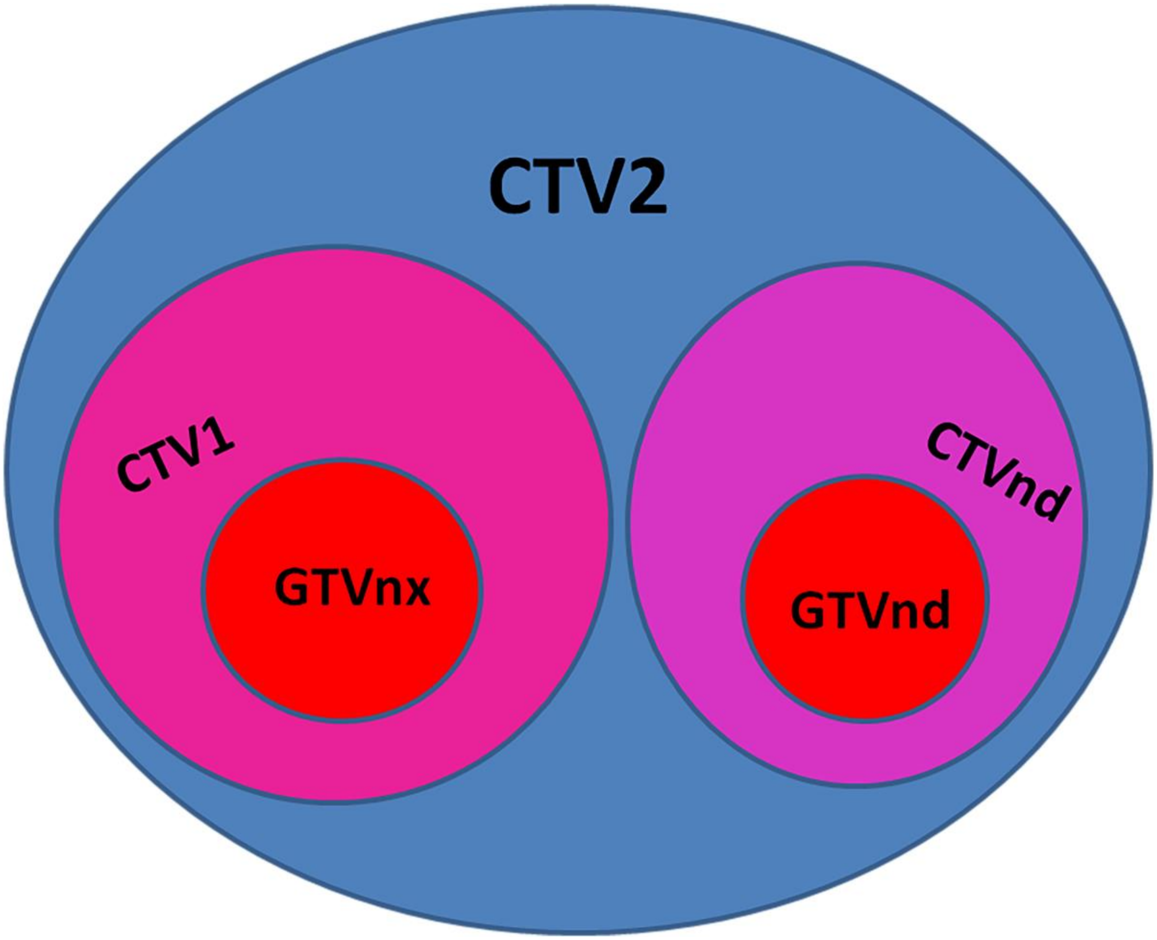

Supplement: Supplementary file 2 [file medi-101-e31187-s002.pdf]
