# Supplementary material for: Efficacy and toxicity of three concurrent chemoradiotherapy regimens in treating nasopharyngeal carcinoma: Comparison among cisplatin, nedaplatin, and lobaplatin
Source: Medicine (Baltimore). 2022 Dec 9;101(49):e31187. doi: 10.1097/MD.0000000000031187 (PMC9750602; doi:10.1097/MD.0000000000031187)
Supplement: Supplementary file 3 [file medi-101-e31187-s003.pdf]

Supplementary table 1. Treatment failure pattern of three concomitant radiochemotherapy regimens

Treatment failure	Cisplatin group	Nedaplatin group	Lobaplatin group
**Locoregional relapse**			
nasopharynx	4	2	4
Lymph node	1	0	3
**Distant metastasis**	10	4	6
Bone	5	3	2
Lung	6	2	2
Liver	6	1	4
Others	1	1	3
Single organ	5	3	1
Multiple organs	5	1	5
**Deaths**	9	2	6
