# Supplementary material for: Efficacy and toxicity of three concurrent chemoradiotherapy regimens in treating nasopharyngeal carcinoma: Comparison among cisplatin, nedaplatin, and lobaplatin
Source: Medicine (Baltimore). 2022 Dec 9;101(49):e31187. doi: 10.1097/MD.0000000000031187 (PMC9750602; doi:10.1097/MD.0000000000031187)

4-Supplementary Figure 2 Hospitalization length (A) and fee (B) between three platinum-based concomitant chemo-radiotherapy regimens. *, $P<0.05$.

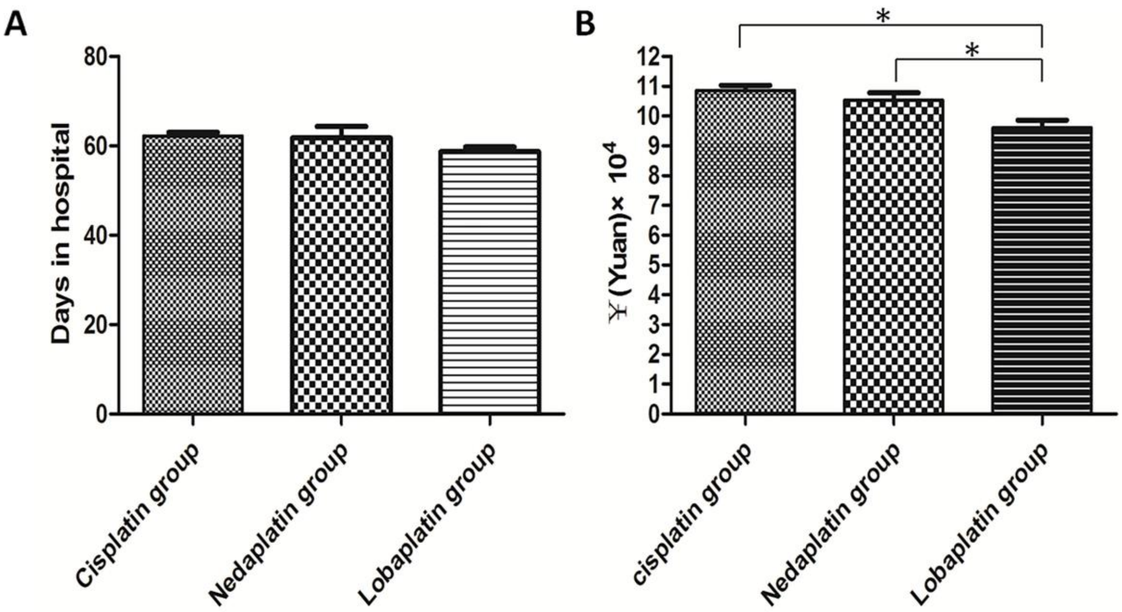

Supplement: Supplementary file 4 [file medi-101-e31187-s004.pdf]
